# The impact of non-lethal doses of pyriproxyfen on male and female *Aedes albopictus* reproductive fitness

**DOI:** 10.3389/finsc.2024.1430422

**Published:** 2024-07-02

**Authors:** Sri Jyosthsna Kancharlapalli, Corey L. Brelsfoard

**Affiliations:** Department of Biological Sciences, Texas Tech University, Lubbock, TX, United States

**Keywords:** non-lethal insecticides, pyriproxyfen, autodissemination, reproductive fitness, vector control

## Abstract

**Introduction:**

Control of the mosquito *Aedes albopictus* is confounded by its behavior due to females preferring to oviposition in small natural and artificial containers that are often difficult to remove or treat with insecticides. Autodissemination strategies utilizing highly potent insect growth regulators (IGRs) have emerged as promising tools for the control of this container-inhabiting species. The intended goal of autodissemination approaches is to use mosquitoes to self-deliver an IGR to these cryptic oviposition locations. Previous studies have focused on the efficacy of these approaches to impact natural populations, but little focus has been placed on the impacts on mosquitoes when exposed to non-lethal doses of IGRs similar to the levels they would be exposed to with autodissemination approaches.

**Methods:**

In this study, the impact of non-lethal doses of pyriproxyfen (PPF) on the reproductive fitness of *Ae. albopictus* was investigated. Female and male *Ae. albopictus* mosquitoes were exposed to non-lethal doses of PPF and their fecundity and fertility were measured. To examine the impact of non-lethal doses of PPF, the expression of the ecdysone-regulated genes *USP*, *HR3*, and *Vg*, which are involved in vitellogenesis, was determined.

**Results:**

Our results demonstrated a significant reduction in female fecundity and in the blood feeding and egg hatching rates upon exposure to non-lethal doses of PPF. Oocyte development was also delayed in PPF-treated females. Furthermore, exposure to non-lethal doses of PPF altered the expression of the genes involved in vitellogenesis, indicating disruption of hormonal regulation. Interestingly, PPF exposure also reduced the sperm production in males, suggesting a potential semi-sterilization effect.

**Discussion:**

These findings suggest that non-lethal doses of PPF could enhance the efficacy of autodissemination approaches by impacting the reproductive fitness of both males and females. However, further research is needed to validate these laboratory findings in field settings and to assess their practical implications for vector control strategies.

## Introduction

*Aedes albopictus* is a vector of dengue, chikungunya, and Zika viruses. Unfortunately, there are no vaccines and therapeutics available to limit the transmission of the aforementioned viruses; therefore, vector control remains one of the only effective measures to limit transmission (1). *Ae. albopictus* is considered a container-inhabiting species, preferring to oviposit in natural and artificial containers found to be associated with human activity. Locating and treating these often abundant and cryptic habitats can present a challenge for mosquito control professionals (2–4). Hence, there is a definitive need for novel approaches to supplement the traditional vector control methods for the effective control of *Ae. albopictus* and other container-inhabiting species.

Autodissemination strategies have been suggested as a potential novel method for the control of *Ae. albopictus* (3, 5–13). Two approaches have been successful in reducing natural populations of *Aedes* spp. The first is autodissemination augmented by males (ADAM), which utilizes laboratory- or factory-reared male mosquitoes that are treated with an insect growth regulator (IGR) and released into the natural environment, where they can directly deliver an IGR to cryptic larval habitats or indirectly by copulating with naturally occurring females (14, 15). The second is autodissemination stations, which rely on attracting females to a station wherein they are then exposed to non-lethal doses of an IGR and leave the station to disseminate the IGR to cryptic larval habitats (5, 7, 10, 11, 13, 16–18).

While autodissemination approaches have been demonstrated to impact mosquito populations, there may also be unintended effects on the male and female mosquitoes exposed to IGRs associated with the application of these approaches. One commonly used IGR that has been used for autodissemination approaches is pyriproxyfen (PPF). PPF is a juvenile hormone analog (JHA). JHAs act similarly to a natural juvenile hormone (JH) in insects. In general, JH and 20-hydroxyecdysone (20E) are two hormones that regulate the developmental, physiological, and reproductive processes in insects. JH interferes with the metamorphosis of the immature stage to the adult stage by retaining the juvenile characteristics in insects, whereas 20E elicits metamorphosis. The antagonistic action of JH and 20E plays an important role in inhibiting important physiological processes, including reproduction and development (19). JH production promotes the primary follicle cells in the ovary to start developing approximately 3 days after eclosion until the production of 20E after a blood meal, which results in oogenesis. In *Aedes* species, 20E plays a major role in upregulating the key genes, such as *EcR* (ecdysone receptor), *HR3* (hormone receptor), *USP* (ultraspiracle), and *Vg* (vitellogenin), that are involved in oogenesis and vitellogenesis. Previous research has demonstrated that exposure to IGRs can disrupt the antagonistic action of these hormones impacting mosquito reproduction, resulting in other unintended physiological effects. The topical application of methoprene, another JHA, downregulated the expression of the ecdysone-regulated genes *USP*, *HR3*, and *Vg*, which are involved in 20E action and oogenesis (20). Furthermore, exposure to PPF has been demonstrated to result in sterilization and fertility effects in adult male and female *Anopheles arabiensis*, *Anopheles gambiae*, *Aedes aegypti*, and *Culex quinquefasciatus* (12, 21–24). In addition, exposure to non-lethal doses of PPF as larvae has been previously demonstrated to increase the susceptibility of *Ae. aegypti* to dengue virus (25).

In this work, experiments were conducted to examine whether non-lethal doses of PPF will reduce the fertility and fecundity of male and female *Ae. albopictus*, respectively. Specifically, these experiments examined the effects of non-lethal doses of PPF on the fecundity and the blood feeding and egg hatching rates of PPF-treated *Ae. albopictus* females and the fertility of males. The results are discussed in the context of the unintended impacts of these non-lethal doses of PPF on male and female mosquitoes associated with the use of autodissemination approaches for mosquito control. The results highlight the potential benefits of non-lethal doses to the effectiveness of autodissemination approaches for mosquito control.

## Materials and methods

### Mosquito rearing and PPF effects on female fecundity, blood feeding, and egg hatching

The *Ae. albopictus* used in the experiments were from a colony established from eggs collected in Lubbock, TX, 33.5846° N, 101.8456° W, and were reared and maintained for ~20 generations in laboratory conditions prior to starting the experiments. The mosquitoes were maintained at 28 ± 2°C temperature and 80% ± 5% relative humidity (RH) in a 16:8 light/dark cycle. The eggs were hatched in a 1:1 solution of deionized water and fermented liver powder (0.6 g/L) (MP Biomedicals, Santa Ana, CA, USA). Larvae were fed a 60-g/L bovine liver powder slurry *ad libitum*. Pupae were collected and placed in 24.5-cm × 24.5-cm × 24.5-cm BugDorm-4S2222 insect rearing cages (MegaView Science Co., Taichung, Taiwan) and provided with a 10% sucrose solution.

For the experiments, Esteem 35WP (Valent Biosciences, Libertyville, IL, USA) was used to treat mosquitoes. Esteem 35WP is formulated with 35% PPF as the active ingredient and was mixed with an inert fluorescent powder (Yu Mingjie pigments, Longdong, Shenzhen, China) to dilute the initial formulations for different adult doses (0.015, 0.03, 0.06, and 0.12 ng). As an untreated control for the PPF dust formulation, the mosquitoes were dusted with only the fluorescent powder, with no Esteem. A total of 30 newly emerged females were treated with each different concentration using a handheld bellow duster (Harris Manufacturing Co. LLC, Cartersville, NC, USA) in a cardboard mailing tube (63.5 mm wide and 20.3 cm long), capped on both ends with No-See-Um netting (Equinox, Williamsport, PA, USA). The mosquitoes were transferred into the rearing cages, with the females fed bovine blood with Na-citrate using an artificial blood feeder and a sausage casing membrane once per week for 3 weeks. A 140-mL Souffle cup (Pactiv, Lake Forest, IL, USA) containing 100 mL deionized water lined with a seed germination paper (Anchor Paper Company, St. Paul, MN, USA) was placed in each cage as an oviposition container. The egg papers were collected for three gonotrophic cycles and the number of eggs counted. The number of blood-fed females was recorded for each cycle. The egg hatching rates were determined for only the first gonotrophic cycle by calculating the mean of three replicate patch counts of approximately 370–1,800 hatched and unhatched eggs. Each treatment consisted of three replicates.

### PPF effects on oocyte development

To visualize the impact of PPF on oocyte development, *Ae. albopictus* females were treated with 0.12, 0.06, 0.03, and 0.015 ng PPF approximately 24 h post-emergence. The treated females were blood-fed 48 h post-exposure and separated into different cages. A total of 10 females from each treatment were collected at 48 and 72 h post-blood feeding. Subsequently, the female mosquitoes were dissected and the ovaries extracted on a small drop of 1× phosphate-buffered saline (PBS). The extracted ovaries were immediately visualized using a Leica S9i stereomicroscope (Leica Microsystems Inc., Buffalo Grove, IL, US), and images were taken using an integrated camera at ×40 magnification.

### PPF effects on the expression of the JH-20E regulated genes

A total of five females were treated with a single dose of PPF at different concentrations (0.015, 0.03, 0.06, and 0.12 ng) and were collected 48 h after blood feeding for each treatment. Each treatment consisted of three replicates. The total RNA was extracted using the Qiagen RNeasy kit following the manufacturer’s instructions (Qiagen, Hilden, Germany). cDNA synthesis was performed using the LunaScript^®^RT SuperMix Kit (#E3010; New England Biolabs, Ipswich, MA, USA) from 1 μg of extracted RNA following the manufacturer’s instructions. The quantitative reverse transcription PCR (qRT-PCR) of the target genes and all the reactions were performed in a 20-μL total reaction volume containing 1 μL cDNA with 1.6 μL primer mix with 10 mM each of a forward and a reverse primer and 10 μL 2× PowerUp™ SYBR™ Green Master Mix (Applied Biosystems, Foster City, CA, USA). All of the samples were analyzed using a qPCR master mix on an ABI 7300 Real-Time qPCR System (Applied Biosystems, Foster City, CA, USA). The temperature profile of the qRT-PCR consisted of 10 min at 95°C, 35 cycles of 30 s at 95°C, 30 s at 54°C, and 1 min at 72°C, followed by a 10-min extension step at 72°C. The relative expression levels of *EcR*, *HR3*, *USP*, and *Vg* were normalized to that of the ribosomal protein S7 (RPS7) using the 2^−ΔΔCt^ method. The primers for the specific genes are listed in **Supplementary Table S1**. Each gene was amplified for four biological and two technical replicates at each time point.

### Male fertility determination

Sperm quantification was performed as described in Ponlawat and Harrington (26). In brief, *Ae. albopictus* males were collected <5–6 h post-emergence and were treated topically with 0.03, 0.06, 0.12 ng of Esteem. After approximately 24 h, 10 mosquitoes were selected, and the testes were dissected in 50 μL PBS using a Leica S9i stereomicroscope (Leica Microsystems Inc., Deerfield, IL, US). Each pool of 10 males was replicated three times. A total of 10 dissected testes were then combined with 150 μL of PBS for a total volume of 200 μL. The testes were gently sheared using a dissection probe, and the sample was mixed well with the P10 pipettor to dislodge any sperm clumps. Eight 5-μL aliquots from the total volume were added to a new microscope slide. Each separate aliquot from the replicate pools was then air-dried for 1–2 h and then fixed with 70% ethanol. Immediately thereafter, the slides were stained with 1 μg/mL DAPI for 10–15 min and the excess stain washed off. The total number of sperm was observed using a Leica fluorescence microscope at ×200 magnification and was counted using ImageJ.

### Statistical analyses

The fecundity, egg hatching, and sperm count data were examined for equality of variance and normal distribution to meet the assumptions of a parametric test. A general linear model with a normal distribution was used to examine for differences in the female fecundity and the number of blood-fed females for the different PPF treatments and gonotrophic cycles. The differences in female fecundity and blood-fed females for each treatment within each gonotrophic cycle were examined using *t*-tests. A repeated measures ANOVA was used to determine differences in the total egg number produced for each cage replicate when combining the data from all three gonotrophic cycles. The egg hatching rates of the females from all three gonotrophic cycles were compared by performing an arcsine square root transformation of the proportion of eggs hatched and an ANOVA. ANOVA was also used to compare the sperm production of males after PPF exposure. Pairwise comparisons of the sperm counts between treatments were performed using *t*-tests. Differences in the gene expression were examined using the non-parametric Kruskal–Wallis multiple comparisons followed by pairwise Wilcoxon tests between the PPF treatments for each gene. All statistical analyses were performed and the graphs designed using JMP Pro 16.

## Results

### Effects of PPF on female fecundity, blood feeding, and egg hatching

**Figure 1:**
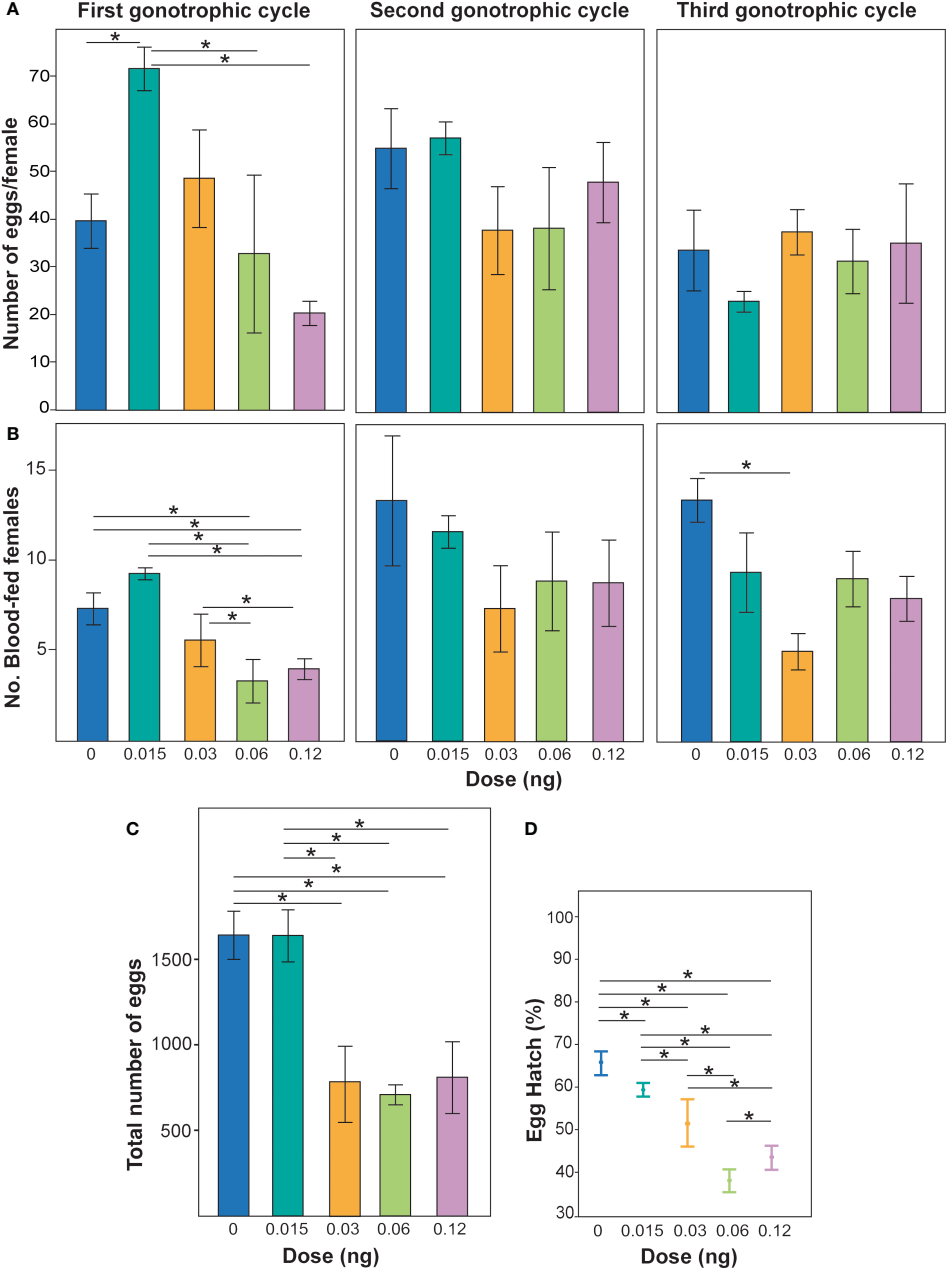
**(A)** Effect of non-lethal doses of pyriproxyfen (PPF) on *Aedes albopictus* fecundity. *Different colored bars* represent the mean ± SEM number of eggs laid per female treated with different concentrations of PPF and untreated females. *Asterisks and lines above each bar* represent significant differences determined using *t*-tests (*p < 0.05). **(B)** Number of blood-fed females post-PPF treatment compared with untreated females over three gonotrophic cycles. *Different colored bars* represent the mean ± SEM number of females seeking blood post-treatment with different PPF concentrations and those with no treatment. *Asterisks with lines above each bar* represent significant differences between treatments determined using pairwise Wilcoxon test (*p < 0.05). **(C)** Total fecundity of females when combining all eggs from blood females over the three gonotrophic cycles and replicates. *Different colored bars* represent the mean ± SEM number of eggs in replicate treatments. *Asterisks and lines above each bar* represent significant differences determined using different *t*-tests (*p < 0.05). **(D)** Percentage of egg hatching collected from females from the first gonotrophic cycle. Data are represented as the mean of three patch counts ± SEM. *Asterisks with lines* in each mean represent significant differences between treatments according to pairwise *t*-tests (*p < 0.05).

Non-lethal doses of PPF were demonstrated to affect the fecundity of *Ae. albopictus* females over the three gonotrophic cycles (*χ* ^2^ = 10.7, DF = 3, *p* = 0.01) (**Figure 1A**). When the effects of PPF dose were examined, a reduction in fecundity was observed (*χ* ^2^ = 3.6, DF = 1, *p* = 0.05), but not for each gonotrophic cycle (*χ* ^2^ = 2.4, DF = 1, *p* = 0.12) (**Figure 1A**). The effect of the interaction of gonotrophic cycle and PPF dose was also associated with a reduction in the fecundity of *Ae. albopictus* females (*χ* ^2^ = 5.6, DF = 1, *p* = 0.01) (**Figure 1A**). Non-lethal doses of PPF were also demonstrated to impact the number of *Ae. albopictus* females that blood fed (*χ* ^2^ = 11.4, DF = 3, *p* = 0.0096) (**Figure 1B**). Examination of the effects of PPF dose revealed an effect of a reduction in the number of females that took a blood meal (*χ* ^2^ = 6.6, DF = 1, *p* = 0.01) and over each gonotrophic cycle (*χ* ^2^ = 5.4, DF = 1, *p* = 0.01) (**Figure 1B**). The effect of the interaction of gonotrophic cycle and PPF dose was not associated with a reduction in the number of females that blood fed (*χ* ^2^ = 0.14, DF = 1, *p* = 0.69) (**Figure 1B**). When combining the egg production over all three gonotrophic cycles, a significant effect of PPF treatment on female fecundity was also observed, where PPF-treated females produced fewer eggs (repeated measures ANOVA: *F* = 6.1, DF = 1, *p* = 0.03) (**Figure 1C**). The data also suggest that, when females were treated with PPF, they produced eggs with significantly lower hatching rates than the untreated females (ANOVA: *F* = 3.9, DF = 4, *p* = 0.04) (**Figure 1D**).

### PPF effects on oocyte development

**Figure 2:**
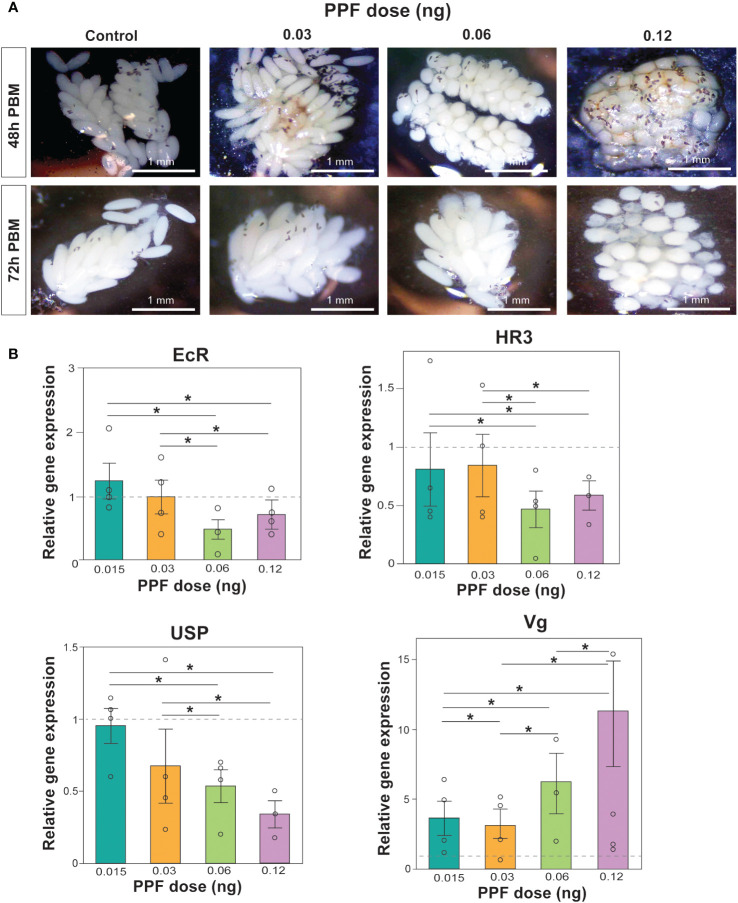
**(A)** Effects of pyriproxyfen (PPF) on *Aedes albopictus* follicle morphology and oocyte development. Labels on the left of the images refer to the number of hours post-blood meal, while the labels on top of the figure refer to the amount of PPF exposure. **(B)** Effects of PPF treatment on the expression of the 20-hydroxyecdysone (20E)-responsive genes after PPF exposure 48 h after a blood meal. Data are presented as the mean ± SEM of four independent replicates. Paired Wilcoxon tests (Bonferroni corrected: *p* < 0.02) were used to determine differences among treatments for each gene. Significant differences between treatments are represented by asterisks and lines above bars.

PPF exposure was also observed to affect ovarian maturation and oocyte development. Images of the dissected ovaries demonstrated the effect of increasing PPF exposure rates on adult females, showing a delay in ovarian maturation and oocyte development compared with the ovaries of the control mosquitoes (**Figure 2A**).

### Impacts of non-lethal doses of PPF on the vitellogenesis pathway

As an additional support for the impact of PPF on the egg production and the hatching rates, the relative expression levels of the 20E-regulated genes involved in vitellogenesis were measured using qRT-PCR. Comparison of the relative expression of *EcR* (Kruskal–Wallis test: *χ* ^2^ = 5.6, DF = 4, *p* = 0.23), *HR3* (Kruskal–Wallis test: *χ* ^2^ = 4.4, DF = 4, *p* = 0.35), *USP* (Kruskal–Wallis test: *χ* ^2^ = 9.4, DF = 4, *p* = 0.052), and *Vg* (Kruskal–Wallis test: *χ* ^2^ = 8.6, DF = 4, *p* = 0.07) showed that the overall gene expression was not impacted by the non-lethal doses of PPF (**Figure 2B**). While there were no significant differences in the overall gene expression, a downregulation of *EcR*, *HR3*, and *USP* and an upregulation of *Vg* were observed when comparing the effects between different doses of PPF 48 h post-blood feeding (**Figure 2B**).

### Effect of PPF on adult male fertility

**Figure 3:**
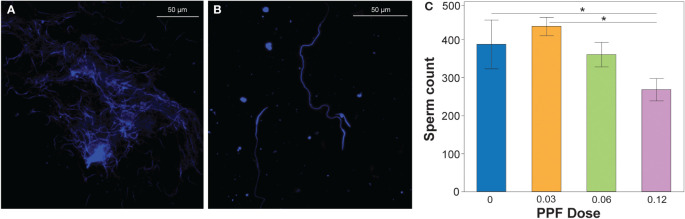
**(A)** DAPI-stained sperm extracted from the dissected testes of an undiluted sample. **(B)** Sperm extracted from the dissected testes of a diluted sample. The number of sperm was counted from the diluted sperm extractions, which were used as a proxy for production by males. **(C)** Effect of pyriproxyfen (PPF) on the sperm production of *Aedes albopictus* males. Paired *t*-tests (Bonferroni corrected: *p* < 0.02) were used to examine the effect of PPF dose on sperm production in males. Significant differences are indicated by a line and asterisk above the sperm counts for the different treatments.

Using the previously described methodology, the sperm production of *Ae. albopictus* males was determined by diluting the pooled male testes dissections (**Figure 3A**) into quantifiable amounts that could be counted on microscope slides (**Figure 3B**). Comparison of all treatments showed no effect of PPF on the fertility and sperm production of *Ae. albopictus* males (ANOVA: *F* = 2.1, DF = 3, *p* = 0.1) (**Figure 3**). However, in the pairwise *post-hoc* comparisons, the mean number of sperm produced by males (mean ± SE = 268.6 ± 43) in the 0.12-ng treatment dose was significantly lower than that of untreated control males (mean ± SE = 387.9 ± 42) and the 0.03-ng treatment dose (mean ± SE = 435.25 ± 24.2) (**Figure 3C**).

## Discussion

The results presented here suggest that non-lethal doses of PPF impact the reproductive fitness of *Ae. albopictus* females. The impact of PPF was observed to be more pronounced in the first gonotrophic cycle than the second and third cycles. This reduction in egg production in the first gonotrophic cycle could be the result of the topical application of PPF. Specifically, PPF could be falling or rubbed off the scales of adult females as they age, consequently decreasing the impact on the fecundity in each subsequent gonotrophic cycle. The observed results are similar to those of earlier studies that reported a reduction in fecundity due to PPF exposure in *Ae. aegypti*, *An. arabiensis*, *An. gambiae*, and *C. quinquefasciatus* (23, 24, 27–29). In addition, this work revealed a non-significant concentration-dependent decline in the number of females that blood fed. The responses of *Ae. albopictus* females to PPF could be due to an undetermined behavioral effect on the blood seeking ability. This is not surprising considering the impacts JH and 20E have on the physiology of insects. It appears likely that, when the JH titers are high in female mosquitoes, they would not be host seeking for a blood meal. This is in accordance with a study reporting that the injection of methoprene and JH III resulted in a lower proportion of females choosing a blood meal over honey compared with the untreated adult female mosquitoes (30).

The observed PPF dose-dependent decline in the egg hatching rates suggests the indirect impact of PPF on the production of viable eggs. Previous studies have demonstrated that, when *An. gambiae* came in contact with the PPF-treated bed nets, ovary development was delayed and females had reduced fecundity (24, 31). JH also affects adult insects by controlling different physiological functions, including vitellogenin, an egg yolk precursor of protein production and synthesis in *Tribolium castaneum* males (32). The JH titers are higher during the previtellogenic stage or 48 h post-eclosion, and the levels drop during blood feeding when the ecdysteroid and *Vg* synthesis begin (33). In the present study, the observed impacts to follicle development post-blood feeding and the reproductive impacts of the non-lethal doses of PPF are expected to be related to the 20E-responsive gene cascade and the associated genes involved in vitellogenesis. These studies demonstrated a non-significant downregulation in *EcR*, *HR3*, and *USP*, which are involved in *Vg* synthesis, possibly due to the inhibition of 20E action by PPF treatment in adult females. Conversely, we observed an increase in the mRNA levels of *Vg* in the PPF-exposed female mosquitoes during the previtellogenic stage, which is responsible for egg yolk protein synthesis in females. These results are similar to those of previous studies that reported an increase in the level of *Vg* in PPF-treated mosquitoes 24 h after a blood meal (20, 34). Conversely, the action of two hormones, JH and 20E, regulate the reproductive maturation in mosquitoes; in this study, the expression levels of the 20E-responsive genes in PPF-treated mosquitoes were significantly lower than those of untreated mosquitoes. This disruption in the normal hormonal responses due to exposure to non-lethal doses of PPF appeared to impact oocyte development and to reduce the potential for viable progeny of the *Ae. albopictus* females.

Previous studies have also focused mainly on the effects of PPF on the fitness of female mosquitoes. In this study, the effect of PPF on sperm production in male mosquitoes was also investigated. A reduction in sperm production in the PPF-treated mosquitoes, when treated with 0.12 ng of PPF, was observed compared with the untreated control. Fertility reduction in the male mosquito population could subsequently impact the viable hatching embryos in females if they were to mate with PPF-exposed males. These results are similar to those of a previous study that observed a significant reduction in sperm production in the PPF-treated laboratory-reared population compared with field populations in Thailand (35). The PPF-induced reduction in male fertility could be interpreted as a semi-sterilization effect. In this work, the males were exposed to 0.12 ng of PPF, the highest dose. Previous work demonstrated that males associated with the use of ADAM approaches were exposed to up to 5 ng of PPF, suggesting that an increased dose of PPF would possibly have an additive effect on lowering the fertility of males (36). This result could be beneficial to increasing the success of autodissemination approaches, particularly ADAM approaches, by inducing a sterilization effect if PPF-treated males were to mate with naturally occurring females. Subsequently, this will impact the population growth rates, in addition to the effects on larvae associated with the dissemination of PPF to larval habitats, and increase the efficacy of autodissemination approaches.

The observed impacts of non-lethal doses of PPF on the reproductive fitness of females and males are a potential benefit to autodissemination approaches. When females and males are exposed to low doses of PPF in autodissemination stations, when males are dosed with PPF as part of an ADAM approach, and when females are indirectly exposed via copulation attempts, these non-lethal impacts could improve the success of these approaches. For example, in either of the aforementioned approaches, females that produce fewer eggs and hatching eggs and males that are semi-sterilized by PPF could have an unintended impact on population growth and consequently have an impact on population control. However, it remains to be seen whether these results observed in laboratory studies can be translated to field studies and measurable impacts as part of autodissemination control approaches. More studies are needed to determine the transfer of PPF to mosquitoes in the field and its persistence on exposed mosquitoes in more natural conditions.

## Data availability statement

The datasets presented in this study can be found in online repositories. The names of the repository/repositories and accession number(s) can be found in the article/**Supplementary Material**.

## Author contributions

SK: Data curation, Investigation, Methodology, Validation, Visualization, Writing – original draft, Writing – review & editing. CB: Data curation, Formal Analysis, Funding acquisition, Investigation, Methodology, Project administration, Resources, Software, Supervision, Validation, Writing – review & editing.
