# Supplementary material for: The impact of non-lethal doses of pyriproxyfen on male and female Aedes albopictus reproductive fitness
Source: Front Insect Sci. 2024 Jul 2;4:1430422. doi: 10.3389/finsc.2024.1430422 (PMC11250599; doi:10.3389/finsc.2024.1430422)
Supplement: Supplementary file 2 [file Table_1.docx]

**Supplemental Table 1.** List of primers for quantitative real-time PCR for the 20E responsive genes (Ahmed et al., 2020).

| **Gene** | **Forward primer** | **Reverse primer** |
| --- | --- | --- |
| Aal-EcR | 5’-GCCACCGTCAGTACAACGAA-3’ | 5’-TGGTAGTAGAGGTATCGCTTGG-3’ |
| Ag-USP | 5’-ACCAGCAACAGAGGAACCAG-3’ | 5’-AATGGACCAAAACCGACCGA-3’ |
| Ag-HR3 | 5’-GATGATTCCGGACGCTCCA-3’ | 5’-GCACACTTTGCAGGGGATGA-3’ |
| Aal-Vg | 5’-TTCGCAAAAGGAACGCCAAG-3’ | 5’-GCATCACGGAAGGTAACCCA-3’ |
